# Nitrogen Balance of Effluent Irrigated Silage Cropping Systems in Southern Australia

**DOI:** 10.1100/tsw.2001.335

**Published:** 2001-11-21

**Authors:** Chris J. Smith, Val O. Snow, Ray Leuning, David Hsu

**Affiliations:** CSIRO Land and Water, Canberra ACT, Australia

**Keywords:** nitrogen balance, volatilisation, piggery effluent-irrigation, silage oats/maize

## Abstract

The nitrogen (N) balance in a double-cropped, effluent spray irrigation system was examined for several years in southern Australia. The amounts of N added by irrigation, removed in the crop, and lost by ammonia (NH_3_) volatilisation, denitrification, and leaching were measured. Results from the project provide pig producers with the knowledge necessary to evaluate the efficiency of such systems for managing N, and enable sustainable effluent reuse practices to be developed. Oats were grown through the winter (May to November) without irrigation, and irrigated maize was grown during the summer/autumn (December to April). Approximately 18 mm of effluent was applied every 3 days. The effluent was alkaline (pH 8.3) and the average ammoniacal-N (NH_4_ + NH_3_) concentration was 430 mg N/l (range: 320 to 679 mg N/l). Mineral N in the 0- to 1.7-m layer tended to increase during the irrigation season and decrease during the winter/spring. About 2000 kg N/ha was found in the profile to a depth of 2 m in October 2000. N removed in the aboveground biomass (oats + maize) was 590 and 570 kg N/ha/year, equivalent to ≈25% of the applied N. Average NH_3_ volatilisation during the daytime (6:00 to 19:00) was 2.74 kg N/ha, while volatilisation at night (19:00 to 6:00) was 0.4 kg N/ha, giving a total of 3.1 kg N/ha/day. This represents ≈12% of the N loading, assuming that these rates apply throughout the season. The balance of the N accumulated in the soil profile during the irrigation season, as 15N-labelled N studies confirmed. The high recovery of the N-labelled N, and the comparable distribution of 15N and Br in the soil profile, implied that there was little loss of N by denitrification, even though the soil was wet enough for leaching of both tracers.

### INTRODUCTION

Commercial pig production requires sound environmental management, particularly in the treatment and reuse of effluent high in nitrogen (N) and salt. When effluent is used for irrigation of crops and pastures, environmental sustainability is judged mainly on its ability to control organic matter, N, other nutrients, salt, and bacteria. For N, sustainable management will only be achieved when the amount exported in the harvested crop and lost to the atmosphere by volatilisation and biological denitrification matches the N-loading (effluent and mineralisation) of the system. If this is the case, the accumulation of large amounts of $NO_3$-N within the soil profile and its subsequent leaching to the groundwater will be prevented. However, such practices would contribute to other off-site effects of ammonia ($NH_3$) emissions and global climate change effects from emissions of nitrous oxide. Piggery effluent is saline (2 to 6 dS/m), so the effluent-irrigated areas must be leached to avoid excessive salt build-up in the root zone. In this paper we report results of a project designed to measure and model losses of N by $NH_3$ volatilisation, denitrification, and leaching from spray-irrigated effluent. The results from the project will assist regulatory authorities in assessing effluent reuse schemes and pig producers in evaluating the cost/benefit of using centre-pivot spray irrigation.

### MATERIALS AND METHODS

The site (lat. 36° 1' S., long. 146° 22'E.) had grown one maize crop (97/98) that was irrigated with 680 mm of treated effluent before the work reported in this paper began. Prior to this time the site had been maintained as unimproved pasture. The soil is classified as a Red Dermosol[1]. Clay content increased with depth from 290 mg/kg soil in the surface 0.1 m to a maximum of 690 mg/kg soil in the layer at 1.15- to 1.8-m depth. The pH (1:5 soil:0.01 M $CaCl_2$) of the surface 0.1 m was 4.75 and increased to about 7.0 at 1.15 to 1.8 m. Additional properties of the surface 0.1 m were (g/kg soil): total C, 16; total N, 1.4; cation exchange capacity ($cmol_{(+)}$ /kg of soil), 9.7.

Rainfall, solar radiation, wind run and direction at 2 m, and temperature and relative humidity were recorded every 15 min with an automatic weather station.

Eight intensive measurement sites were established within the area irrigated by the centre pivot (600-m radius). At each site we installed soil solution samplers (0.25-, 0.45-, 1.0-, and 1.4-m depth), neutron probe access tubes, and Br and $^{15}N$ subplots (3.4 $m^2$). Crop growth and changes in mineral N in the soil were monitored near each of these sites. Oats were grown during the winter/spring (May to November) and maize during the summer months (December to April). Details of sowing and harvesting dates, irrigation period, and amounts over the period are presented in Table 1. All of the aboveground biomass was harvested for silage. Irrigation with treated piggery effluent started in mid-December after emergence of the summer crop (Table 1). The irrigator was set so that it took approximately 72 h to complete

**TABLE 1**
**Cropping Sequence, Management and Irrigation Details**

| Crop | 1998 Oats | 1998/99 Maize | 1999 Oats | 1999/2000 Maize | 2000 Oats |
|---|---|---|---|---|---|
| Sowing date | 01 June 1998 | 01 Dec 1998 | 25 April 1999 | 29 Nov 1999 | 20 May 2000 |
| Cultivar | Echidna | Hycorn 72 | Echidna | Hycorn 75 and Pioneer 3394 | Echidna |
| Sowing rate | 100 kg/ha | 25 kg/ha | 100 kg/ha | 25 kg/ha | 100 kg/ha |
| Row spacing | 0.30-m rows | 0.76-m rows (85,000 seeds/ha) | 0.30-m rows | 0.76-m rows (85,000 seeds/ha) | 0.30-m rows |
| Fertiliser/spray at sowing | 80 kg/ha Triphos | 3 l/ha Stomp 2 l/ha atrazine (08 Dec 1998) | | 1.5 l/ha Dual Goal 2.4 lt/ha Gesaprim (05 Dec 1999) | |
| Irrigation start | N/A | 08 Dec 1998 | N/A | 05 Dec 1999 | N/A |
| Irrigation amount | 18-mm abattoir water (25–27 May 1998) | 558 mm; 72-h cycle, 18 mm/cycle | 28-mm abattoir water (30 April–04 May 1999) | 144-mm abattoir water; 472-mm effluent. | N/A |
| Irrigation finish | N/A | 13 Mar 1999 | N/A | 31 March 2000 | N/A |
| N applied (kg/ha) | 15.5 | 2510 | 24 | 2248 | N/A |
| Harvest date | 02 Nov 1998 | 10 April 1999 | 06 Oct 1999 | 13 April 2000 | 10Oct 2000 |
| Silage yield (wet weight) | 29.6 t/ha | 60.8 t/ha | 26.7 t/ha | 44.8 t/ha | 44.4 t/ha |
| Fallow treatment | Crash grazed; residue burnt 26 Nov 1998 Site deep ripped to 0.2 m on 27 Nov 1998; harrowed twice | Crash grazed | Crash grazed | Crash grazed | Crash grazed |

an irrigation cycle and approximately 18 mm of irrigant was applied in each cycle. Effluent characteristics are given in Table 2.

Samples of the crops (oats and maize) were collected from near the eight measurement sites at approximately 3-week intervals throughout the growing season. Sampling consisted of cutting all the aboveground biomass from 1 m of three adjacent rows. The area sampled was 0.9 $m^2$ for the oats and 2.25 $m^2$ for the maize. The plant material was divided into green leaf, senescent leaf, stem, and head fractions, dried at 60°C, and weighed to determine dry weight. The dried material was ground to pass a 0.42-mm sieve and retained for total N analysis. Subsamples of the plant material were ground to pass a 0.42-mm sieve and total N content was determined on a Leco CHN analyser (Model CHN-1000). Biomass and total N data are expressed on an oven-dry weight basis.

At the end of each crop, soil cores (≈40 mm i.d.) were taken near each of the eight sampling sites. The cores were sectioned into 0.05-m intervals to 0.2-m depth, 0.1-m intervals from 0.2- to 0.5-m depth, and 0.2-m intervals from 0.5- to 1.9-m depth. After thorough mixing of the soil samples, $NH_4^+$ and $NO_3^-$ (including $NO_2^-$) were extracted by shaking the equivalent of 10 g of dry soil with 100 $cm^3$ of 2 *M* KCl for 1 h[2]. The suspensions were filtered and the filtrate stored frozen until analysed. Ammonium was measured by reacting with hypochlorite liberated from dichlor-isocyanurate under alkaline conditions. The absorbance was measured at 660 nm using a dual channel segmented flow analyser[3]. $NO_3$ was determined by quantitative reduction to nitrite by cadmium metal in a packed-bed cadmium column using a segmented flow analyser[3]. All results were expressed on a oven-dry weight (105°C) basis.

Bromide (Br) and $^{15}N$-labelled $(NH_4)_2SO_4$ (10 atom %) were applied uniformly to three microplots in the two maize crops (1999 and 2000) and to the 2000 oat crop to allow estimates of losses by denitrification and leaching. The unconfined microplots were 3.4 $m^2$ (1.5 × 2.25 m) in the maize and 1 $m^2$ in the oats. The $^{15}N$ and Br solution was applied as a liquid to the soil surface of the maize plots using a hand sprayer at the rate of 800 $cm^3/m^2$ that added 5 $gN/m^2$ (10 atom %) and 12.7 g $Br/m^2$. Spray marker was applied with the solution to assist uniform application onto the soil surface. For the oats, the tracer was applied by injecting 2 $cm^3$ of solution on a 5- × 5-cm grid[4]. At harvest, only 0.75 m of the two centre rows of maize was harvested, whereas 0.5 m of the centre two rows was harvested in the oats. Two soil cores (≈40 mm i.d.) were taken from the centre area of each microplot and sectioned as for the mineral N samples. Depth of sampling was to 1 m at harvest of the 1998/99 maize crop and to 2 m at harvest of the 2000 oat crop. The $^{15}N$ labelled and Br solution was applied to the 1998/99 maize crop on January 15, 1999 and February 10, 1999, to the 1999/2000 maize crop on December 14, 1999, and to the 2000 oat crop on June 8, 2000.

$NH_3$ volatilisation was measured using micrometeorological techniques during two intensive field campaigns (January 11 to January 29, 1999; February 1 to February 21, 2000). Atmospheric measurements were made at two sites positioned 65 m from the centre of the pivot irrigation boom. In 1999, the instruments and air intakes were positioned at 1.9, 2.9, and 3.9 m aboveground and were lowered and covered during the times that the irrigation boom passed over the sites.

For the second field campaign in February 2000, the sampling strategy was revised to better estimate $NH_3$ lost to the atmosphere, because water droplets containing $NH_3$ evaporate when they are ejected from the boom sprayers. Standard 1-D methods were used to measure the $NH_3$ emission for the effluent irrigated area, whereas a mass balance approach was employed when the irrigation boom was within an arc of 40° of the sampling masts. This required the measurement of profiles of $NH_3$ concentrations both upwind and downwind of the irrigation boom. The instruments and air intakes were positioned at 0.5, 1.0, 1.8, 3.58, 4.56, 6.06, and 9.21 m aboveground. The measurement stations were shut down, sampling towers lowered, and instruments covered during the times that the irrigation boom passed over the sites. Ultrasonic anemometers measured turbulent wind velocities, and rapid fluctuations in $CO_2$ and $H_2O$ concentrations were measured using open-path infrared analysers. Fluxes of water vapour and carbon dioxide were then computed for half-hourly periods using the eddy covariance technique. Profiles of wind speed, atmospheric temperature, and relative humidity were measured over 30-min averaging periods, as well as net radiation, soil temperature, and atmospheric pressure. $NH_3$ and water vapour mixing ratios averaged over 30 min were measured by Fourier Transformed Infrared Spectroscopic (FTIR) instrumentation[5]. The measurements were combined to calculate $NH_3$ volatilisation fluxes using micrometeorological theory.

## RESULTS AND DISCUSSION

### Climatic Conditions During the Measurement Period

In evaluating the results, it is important to know how climatic conditions during the experiments relate to long-term averages.

**TABLE 2**
**Selected Characteristics of Effluent from Waste Streams**

| Source | pH | EC (dS/m) | TDS† (mg/l) | Ammonium-N (mg/l) | Inorganic-N (mg/l) |
|---|---|---|---|---|---|
| Abattoir | 7.4 ± 0.1 | 1.8 ± 0.02 | 1130 | 70 ± 3.7 | 86 ± 5.8 |
| Piggery | 8.1 ± 0.3 | 5.6 ± 0.9 | 3580 | 430 ± 85 | 450 ± 109 |

† Total Dissolved Salts (TDS; mg/l) = 640 × EC (dS/m); inorganic N was the major N source in the treated effluent.

More than 100 years of rainfall data are available from the SILO Point Patched Dataset[6] for Corowa, which was within 5 km of the site[7]. Although rainfall at the measurement sites can be different from that measured in Corowa, especially during the summer, there was a general linear relationship between the two (slope 0.97; $r^2 = 0.7$). Because of the good agreement, we used the historical data to indicate the general relationship between rainfall during the measurement period and the long-term average (Fig. 1). With few exceptions, monthly rainfall was below average between March 1998 and July 1999; after that, there were many months both above and below average by more than 20 mm, with a slight above average trend (11 compared with 9). The highest monthly rainfall was measured in August 1999, which was 70 mm above average and would have a significant effect on leaching. Rainfall above average by more than 20 mm was recorded for August, September, and November 2000.

### Crop Growth and N Accumulation

The biomass of the oats increased rapidly during the spring and at harvest: 9.13 ± 1.83, 12.6 ± 1.8, and 12.9 ± 1.8 t/ha was removed from the site in 1998, 1999, and 2000, respectively. The oat crops removed 200, 260, and 270 kg N/ha; whereas the maize crops removed 390 and 310 kg N/ha (Fig. 2). The lower N removal by the 1999/2000 crop may be due to increased soil salinity that reduced the growth of the maize. Over the measurement period (May 98 to December 2000), 72 t/ha of biomass containing 1430 kg N/ha was exported from the site in silage. These data indicate that for a double-cropping cycle (oat and irrigated maize) 570 to 590 kg N/ha was exported, equivalent to 23 to 26% of the N applied in the effluent.

### $NH_3$ Volatilisation

Volatilisation of $NH_3$ was highest during and soon after irrigation, consistent with high soil pH values that increase the partial pressure of $NH_3$-N at the soil surface. $NH_3$ fluxes were variable and were lower at night when there was less energy available for evaporation. Average volatilisation rates during daytime (6:00 to 19:00) ranged from 2.7 to 1.2 kg N/ha/day, while volatilisation at night (19:00 to 6:00) varied from 0.1 to 0.4 kg N/ha. The daily emissions (excluding $NH_3$ emission from the spray droplets) were 3.1 kg N/ha/day in 1998/99 and 1.3 kg N/ha/day in 1999/00. Volatilisation from the boom was estimated to be 1.9 kg N/ha/day in February 2000. If we assume that the emission from the

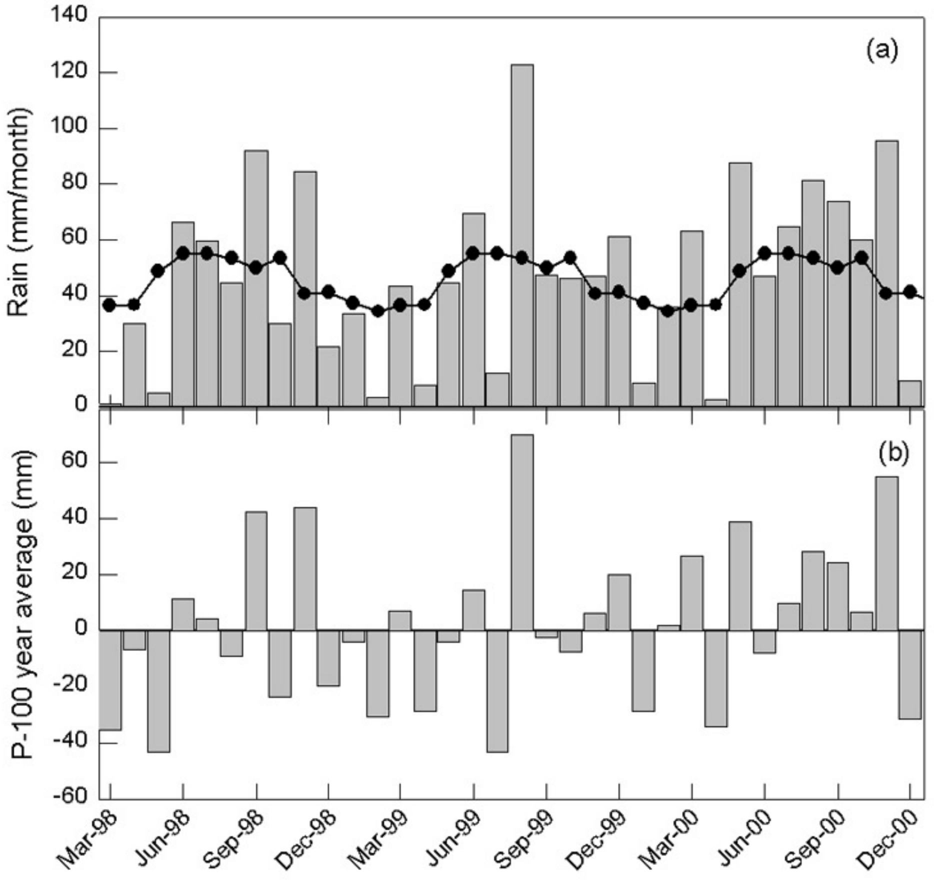

**FIGURE 1.** Monthly rainfall (P; vertical bars) at the effluent irrigated site, average 100-year monthly rainfall (●), and the difference between monthly rainfall and the 100-year average rainfall from March 1998 to December 2000 (100-year average from the SILO Point patched dataset[6]).

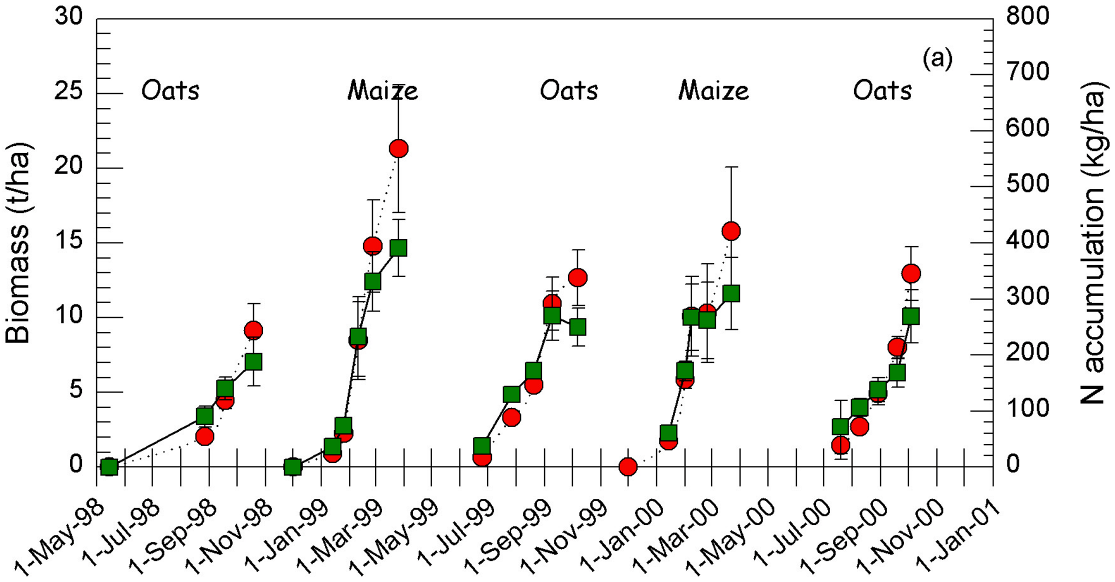

**FIGURE 2.** Averaged biomass (●) and total N (■) accumulation in the tops of the oats and maize. Vertical bars represent the standard error ($p$ = 0.05) of the mean at each sampling time.

boom was the same for both years, the total emission of $NH_3$ was estimated to be 462 and 298 kg N/ha for the 98/99 and 99/00 irrigation seasons, respectively, which is equivalent to about 12 to 18% of the N loading.

### Soil Mineral N

Ammonium was the predominant N species in the effluent (Table 2). Once applied to the soil, the ammonium was rapidly nitrified to $NO_3$ as evidenced by the low ammonium but high $NO_3$-N values in the soil. There was an increase over time in the $NO_3$-N concentration in the surface 1 m between May 1998 and May 1999, which is attributed to the below-average rainfall during this time. After that time, the concentrations decreased in the surface but increased at depth (>1 m), suggesting that $NO_3$ was leaching beyond the root zone of the crop (Fig. 3). This tended to occur during the winter months when rainfall significantly exceeded potential evapotranspiration.

The soil contained about 2250 ± 630 kg of mineral-N/ha to a depth of 1.7 m after the oats had been sown in May 1998 (Fig. 3). The storage increased to a maximum of about 4740 kg N/ha by May 1999, shortly after the maize crop had been harvested. The net increase in mineral N storage between May 1998 and May 1999 mainly occurred in the 0.5- to 1.1-m layer, with little change in the surface 0.5 m and in the 1.1- to 1.7-m layers. In November 1998 it was not physically possible to sample all sites to 1.7 m because the soil was dry. Consequently, data below 1.0 m are biased towards the wetter sites. No effluent was applied during the winter period, but there was net mineralisation of the soil organic matter. Between May 1999 and October 2000, mineral N stored to 1.7 m decreased by ≈2995 kg N/ha; the net decrease was 1950 kg N/ha in the surface 0.5-m layer and 837 kg N/ha in the 0.5- to 1.1-m soil layer. The decrease in the amount of mineral N stored in the profile coincided with the period of above-average winter/spring rainfall (Fig .1). As shown above, uptake and export in the oats and maize accounted for 840 kg N/ha, and 2248 kg N/ha (Table 1) was added in effluent (1999/2000). These data suggest that either leaching or denitrification was responsible for the large decline in mineral N.

### Mineral N Losses through Leaching and Denitrification

There is evidence of some leaching of mineral N beyond the root zone of the crops, as evidenced by the downward movement of $NO_3$-N during the winter/spring period of 1999 (Fig. 3). Because maize uses more water than rainfall plus irrigation, there is a trend of net accumulation of $NO_3$-N during the irrigation season (summer/autumn) in the upper soil layers with limited downward movement. During the winter/spring (non-irrigation period) concentration decreased in the surface soil (Fig. 3) as a result of leaching, crop uptake, and denitrification. However, N balance calculations suggest that the amount of N applied in the effluent and from the breakdown of organic material is far greater than the amount removed in the crop or lost from the system by volatilisation.

The trend of mineral N accumulation during the irrigation season is confirmed from the distribution of $^{15}N$-labelled material in the soil profile (Fig. 4). These data show that most of the $^{15}N$ was recovered in the upper part of the soil profile when applied to the 1998/99 maize crop (Fig. 4a, b). In contrast, the $^{15}N$-labelled material moved to a greater depth during the wet winter of 2000 (Fig. 4c,d), compared with that applied during the irrigation season. Between 94 and 108% of the $^{15}N$ applied in January and February 1999 was recovered in the soil and plant. The high recovery of the $^{15}N$ during the irrigation season confirms

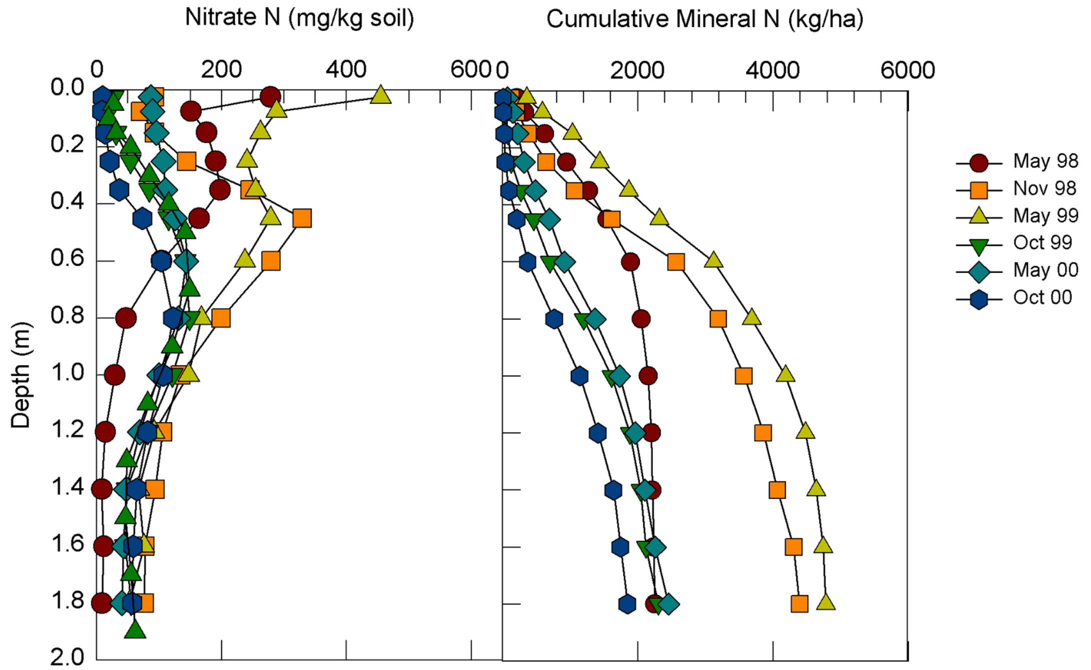

**FIGURE 3.** Mineral N values in the 0- to 1.9-m soil layer at sowing of the oats (May 98), after harvest of the oats (November 1998; October 1999, October 00), and after harvest of the maize (May 1999, May 00). The site was irrigated from December through March each year with treated effluent (see Table 1).

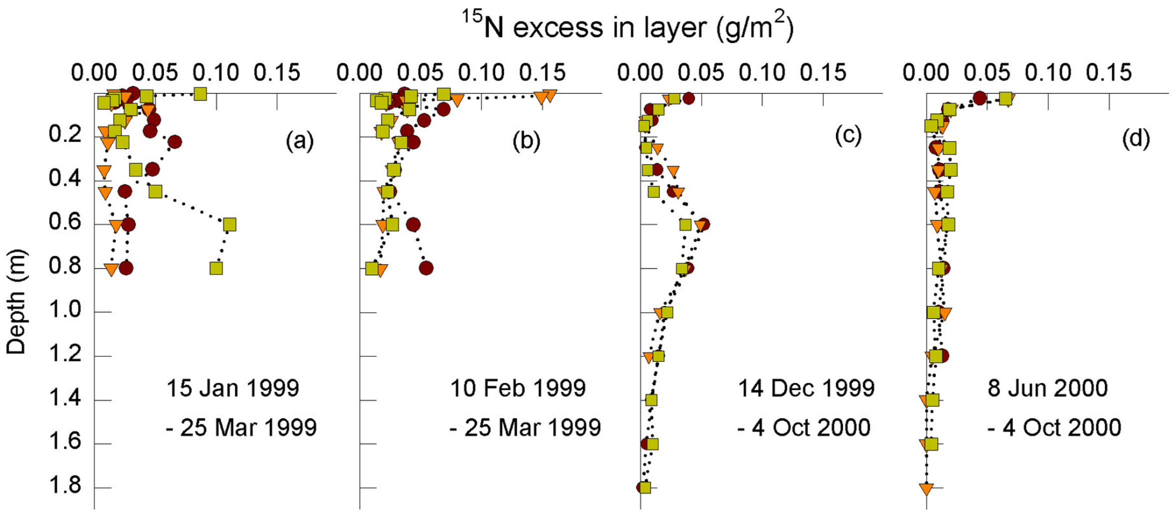

**FIGURE 4.** Distribution of $^{15}N$ in the soil profile of the three subplots at harvest of the 1998/99 maize crop (a, b) and at harvest of the 2000 oat crop (c, d). $^{15}N$ labelled $(NH_4)_2SO_4$ and Br were applied on (a) January 15, 1999, (b) February 10, 1999, (c) December 14, 1999 and (d) June 8, 2000. The site was irrigated from December to March with treated piggery effluent (Table 1).

that there was little loss of mineral N during this period. Although data on this is not shown, the Br had a similar distribution to $^{15}N$ in the soil profile. The shape of the $^{15}N$ distribution with depth (Fig. 4c) indicates that the tracer applied in December 1999 had been leached to about 0.8 m by October 2000. The high recovery of the $^{15}N$-labelled N, and the comparable distribution of $^{15}N$ and Br in the soil profile, implied that there was little loss of N by denitrification, even though the soil was wet enough for leaching of both tracers. It is concluded that leaching was the dominant loss mechanism on these sites.

### CONCLUSIONS

These data show that high-strength effluent, when used as an irrigant, resulted in an N application that exceeded the amount removed by harvesting the crops for silage. $NH_3$ volatilisation during effluent application and from the soil-plant surface was not sufficient to remove the excess N. Furthermore, the large accumulation of $NO_3$ in the soil profile suggests that biological denitrification could not remove the excess N. There was evidence of leaching of $NO_3$ to depth in the profile, confirmed by the distribution and high recovery of $^{15}N$ in the soil profile. Application of N in effluent applied at rates that exceed the crop requirements substantially increases $NO_3$ leaching beyond the root zone. However, land application schemes can become sustainable by either reducing the nutrient concentration of the effluent applied or by applying nutrients at a rate that matches the crop N demand and applying supplemental irrigation.

### ACKNOWLEDGMENTS

This research was partially supported by the Pig Research and Development Corporation.

| | | |
|---|---|---|
| Received: | July | 26, 2001 |
| Revised: | October | 15, 2001 |
| Accepted: | October | 29, 2001 |
| Published: | November | 21, 2001 |
